# Objective selection of bone mimetic materials using impact microindentation

**DOI:** 10.1016/j.jor.2025.11.038

**Published:** 2025-11-29

**Authors:** Lucas R. Budd, Rachana S. Vaidya, Babak Jahani, Saideep Nakka, Alexander Proctor, Peter T. Burks, Blake K. Montgomery, Simon Y. Tang

**Affiliations:** a Department of Orthopaedic Surgery, Washington University School of Medicine in St. Louis, MO, 63110, USA; b Department of Biomedical Engineering, Washington University School of Medicine in St. Louis, MO, 63130, USA; c Medtronic Inc. 4620 N Beach St, Fort Worth, TX, 76137, USA; d Active Life Scientific, Inc, Santa Barbara, CA, USA

**Keywords:** Bone mimetic selection, Impact microindentation, Bone hardness, Surgical training, Implant testing

## Abstract

**Background:**

Research and development for orthopedic medical devices, implants, surgical technique, and surgical education all require the appropriate selection of bone mimetic materials to align with the physiologic conditions. There is a critical need for an objective measure of material strength that can be collected, practically, on both bone mimetic materials and living human bone to improve mimetic material selection.

**Methods:**

This study stratifies commonly used synthetic materials in orthopedic research, as well as animal and human bone, using impact microindentation. The impact microindentation is performed with the OsteoProbe, an FDA-approved handheld medical device that can quantify material strength via the Bone Material Strength index in living human bone and mimetic materials non-destructively and in situ. Categories of Low, Mid, and High are created in reference to published living human tibia Bone Material Strength index ranges. All materials tested are classified into these categories for streamlined bone mimetic material selection.

**Results:**

Nine mimetic materials fell within the Low category. Two mimetic materials were classified as Mid and two as High. The male human cadaver cranium, radius, and femur fell within the High category; the male spine was classified in the Mid hardness category. In female cadaver tissue, the spine corresponded to the Low hardness category, the radius to Mid, and the femur to High. The ovine skull fell within the Mid category. Both the porcine thoracolumbar spine and bovine scapula fell within the High category.

**Conclusion:**

This unified scale of material strength allows for the comparison of human bones, animal bones and synthetic materials. By stratifying bone mimetics into three categories (Low, Mid, and High) of material strength based on healthy reference interval of living human Bone Material Strength index values, this study introduces a simplified framework to guide targeted material selection in orthopedic research and surgical training.

## Highlights

• Quantified the hardness of bone mimetic, human cadaver, and animal tissues using impact microindentation.
• Developed Low, Mid, High classification of material strength based on living human impact microindentation values.
• Streamlined selection of mimetic materials with improved alignment between experimental models and physiologic bone strength.

## 1 Introduction

Bone quality is a crucial factor for the success of orthopedic surgery involving instrumentation, as poor bone quality can lead to serious complications such as fractures, hardware instability, and incomplete healing.^1^, ^2^, ^3^ Further, mis-matched mechanical performance between hardware and bone, even in healthy bone, can lead to sub-optimal outcomes. Surgeons training to perform orthopedic procedures, and researchers developing medical technologies for musculoskeletal tissues, commonly rely on standardized and reproducible bone mimetic materials.^4^, ^5^, ^6^, ^7^ However, because bone-mimetic materials are compositionally homogeneous, they fail to fully capture the natural variability in human bone quality and strength. Since bone quality varies greatly with age, sex, and comorbidities^8^ there is a critical need to select bone mimetic materials that properly align with the intended application and are generalizable to the target population. Until recently, there has not been a consistent method to measure the mechanical function of both mimetic and living bone in the same geometric configuration and scale that allows the direct comparison of the two.

The conventional standards for assessing material and mechanical properties such as fracture toughness^9^, ^10^, ^11^ and hardness rely on engineering tests.^12^ These destructive mechanical testing methods, such as bending, tension, compression, shear, indentation, and fracture toughness, have been employed to characterize the mechanical competence of the bone matrix.^13^, ^14^, ^15^, ^16^ Each of these methods leverages different mechanical principles and assumptions to define material properties. These methods require specialized equipment and sample modifications including polishing and cutting into standardized geometries. While bone mimetic materials can be characterized with these engineering methods, these tests of mechanical behavior cannot be conducted in the living human. Additionally, human cadaveric tissues used in clinical training and orthopedic research are not regularly evaluated with these robust engineering methods. For example, the quality of donor bone tissue is frequently inferred from age or medical history; however, these parameters function only as indirect proxies and do not directly assess the mechanical integrity of the patient's bone. Because these tests are often destructive, it is not possible to both measure the mechanical behavior of the bone and ensure that it remains intact for the intended training or research purpose.

Clinical measures of bone health, including x-ray and DXA, offer some insight into bone quality. However, these imaging modalities underperform at capturing bone strength relative to engineering methods and do not strongly correlate with mechanical properties such as fracture toughness.^17^^,^^18^ For example, x-ray and DXA can robustly measure gross bone geometry and bone mineral density. However, these are only a subset of bone characteristics that contribute to the overall material properties.^19^, ^20^, ^21^ The strength of bone is derived from factors across multiple length scales. Though some microstructural factors such as cancellous bone mass density, cortical porosity, and mineralization can be measured clinically; others, particularly at small length scales, such as the composition of the organic matrix and the orientation of these proteins cannot be captured in radiographic imaging.^22^^,^^23^ Therefore, physicians often rely on subjective measures of bone quality such as tactile feedback.^24^ The absence of a universally accepted quantitative metric for bone strength has led to inconsistent classification of bone as being “good” or “poor” quality, further obscuring the selection of bone mimetic materials that match bone hardness. Determination of bone quality is perhaps most critical when bone does not physically behave in accordance with radiographic imaging and patient history, such as in pathologies with discordant bone quality deficits.^25^ Despite widespread idiomatic use among clinicians, subjective assessments of bone quality can lead to inconsistency and suboptimal outcomes in orthopedics.^26^ There is a need for guided bone mimetic material selection using an objective measure that captures fundamental material strength properties and can be implemented in vivo for humans.

Impact microindentation (IMI) has been validated as an effective and non-destructive method for assessing material strength.^27^ IMI measures how resistant the bone tissue is to indentation and serves as a proxy for its material-level strength and toughness. IMI is advantageous due to its non-destructive nature and minimal requirements for sample preparation. The currently commercially available device that performs IMI, the OsteoProbe, is an FDA-Approved medical device that uses IMI to quantitatively measure the mechanical competence of bone. The device performs an indentation on the micron length scale by measuring the depth penetrated by a sterile tip at the cortical bone surface. The output of OsteoProbe is the Bone Material Strength index (BMSi) which is a calibrated scale that is inversely related to the depth of tip indentation into the bone. Therefore, lower scores equal deeper indentation while higher scores denote a shallower indentation. BMSi measurements are associated with whole bone strength,^28^, ^29^, ^30^, ^31^ bone fracture toughness^27^ and indentation hardness such as Rockwell and Vickers tests.^32^ The BMSi captures underlying properties of bone that result in strength such as mineralization, collagen cross linking, and cortical porosity.^33^ In one case, traditional preoperative risk assessment failed to identify an individual with poor bone strength and resulted in a spiral fracture during a cementless total hip replacement while the OsteoProbe indicated low bone resistance.^34^

Despite the utility of measuring bone strength via the OsteoProbe, there has yet to be a comprehensive evaluation of bone mimetic materials using the device. The goal of this study is to use the OsteoProbe medical device to assess the strength of commonly used bone mimetic materials and physiologic bone tissue to guide synthetic material selection. Defining mechanical performance based on Osteoprobe measurements will enhance the selection of accurate bone mimetic materials. This evaluation is especially important for selecting materials that are the most aligned with the BMSi at the site of surgical instrumentation. Using a previously published reference interval of healthy living human tibia BMSi values, categories of material strength can be classified to simplify and streamline bone mimetic material selection while using objective measures.^35^ These measures were then compared to BMSi values obtained from the human cadaver radius, spine, and femur, and animal skull, spine and scapula.

## 2 Methodology

### 2.1 Sample selection

The OsteoProbe quantified the BMSi of synthetic bone mimetic materials, animal models, and human cadaveric models. Thirteen commonly used synthetic bone mimetic materials from 4 different suppliers were used to establish the hardness/quality range of commonly used synthetic bone materials. These synthetic materials included 1 sample from Stratasys, 1 sample from BoneSim, 4 samples from Sawbones, and 7 samples from PHACON. Human cadaver bones were obtained from multiple tissue banks (NDRI, Science Care, AGR). Specimens with fractures or orthopedic hardware at IMI testing sites were excluded. All cadaver and animal model samples were stored in −80°C, and then gradually thawed to 20°C before testing. The average age of the male cadavers was 73.7 years (95 %CI: [67.8, 79.6]). The average age for the female cadavers was 71.5 years (95 %CI: [68.3, 74.7]). The average weight of the male cadavers was 207.6 lbs (95 %CI: [201.7, 213.5]); for the female cadavers 178.6 lbs (95 %CI: [159.8, 197.4]). The donor tissues included male spine (L4 vertebrae) (n = 17), male radius (n = 16), male femur (n = 20), female spine (L4 vertebrae) (n = 52), female radius (n = 56), and female femur (n = 67). One male 55 y/o cranium was measured. Previously reported living human male (n = 197) and female tibia (n = 282) were reanalyzed and included here for material recommendation.^35^

### 2.2 OsteoProbe testing

For all synthetic materials, except Stratasys (1) and Sawbones Skull Model (3) a minimum of 5 sample replicates were used, with 5 indentations on each sample. The average and 95% CI are reported for all synthetic and biologic tissue BMSi measurements. The bone mimetic materials were parsed into categories of low, mid and high based on the observed BMSi range in healthy human tibia (48.1–101.4) from a previously reported study.^35^ The mid category corresponds to the middle third of the range (65.9–83.6), the low category includes values below 65.9, and the high category includes values above 83.6.

To best capture bone strength measurements in an anatomical site of interest, several indentations were conducted. A minimum of 5 indents, or as many that are feasible within a flat homogenous region of bone, were used to ensure representative BMSi values were obtained. Spine, radius, and femur BMSi scores were obtained from 8 indentations; the cranium score was obtained from an average of 5 BMSi scores with 5 indentations each score. Cranium measurements were collected at frontal and parietal locations. Radius measurements were collected at the anterior distal radius. Femur measurements were taken at the midshaft. Spine measurements were taken at the pedicle. Tibia measurements were obtained on the anterior aspect of the tibia midshaft.

The BMSi of ovine skull, porcine thoracolumbar spine, in addition to bovine scapula were measured. 36 indentations were conducted on the ovine skull to generate 6 BMSi measurements. 27 indentations were conducted on the porcine spine to generate 5 BMSi measurements. 34 indentations were conducted on the porcine spine to generate 5 BMSi measurements.

## 3 Results

For the human cadavers, the male and female femur BMSi values were 92.4 (95 %CI: [91.0, 93.9]) and 90.0 (95 %CI: [88.7, 91.4]). The male and female radius BMSi values were 87.0 (95 %CI: [82.7, 91.2]) and 78.9 (95 %CI: [76.2, 81.5]). The male and female spine BMSi values were 68.6 (95 %CI: [66.3, 70.9]) and 64.2 (95 %CI: [61.2, 67.2]) (Table 1). The male cranium BMSi values were 95.0 (95 %CI: [93.7, 96.4])

**Table 1:** Human cadaver and animal bone measures relative to the tiers of bone mimetic material.

	Biological Tissue	BMSi	95 % CI	N	Biomimetic Category Recommendation
**Human Cadaver**	Male Spine	68.6	[66.3, 70.9]	17	Mid
	Male Radius	87.0	[82.7, 91.2]	16	High
	Male Femur	92.4	[91.0, 93.9]	20	High
	Male Cranium	95.0	[93.7, 96.4]	1	High
	Female Spine	64.2	[61.2, 67.2]	52	Low
	Female Radius	78.9	[76.2, 81.5]	56	Mid
	Female Femur	90.0	[88.7, 91.4]	67	High
**Animal Model**	Ovine Skull	75	[74.0, 76.0]	1	Mid
	Porcine Thoracolumbar Spine	88	[85.7, 90.3]	1	High
	Bovine Scapula	87	[85.0, 89.0]	1	High

The ovine skull BMSi value was 75 (95 %CI: [74.0, 76.0]). The porcine thoracolumbar spine BMSi value was 87 (95 %CI: [85.0, 89.0]). The bovine scapula BMSi value was 88 (95 %CI: [85.7, 90.3]) (Table 1).

**Fig. 1:**
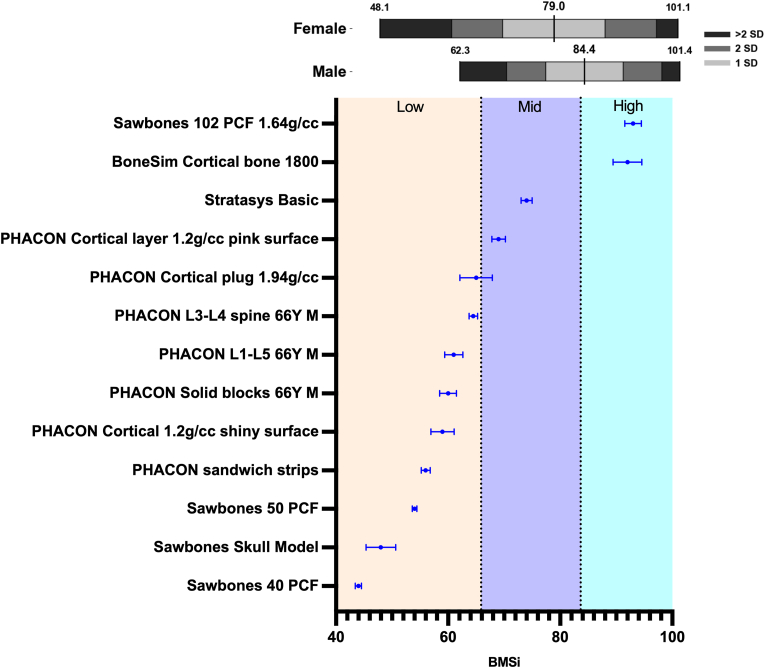
BMSi ranges of healthy living male tibia (min = 62.3, max = 101.4, mean = 84.4, SD = 6.9) and female tibia (min = 48.1, max = 101.1, mean = 79.0, SD = 9.1) from a previously reported study 34. Bone mimetic materials were placed into BMSi categories of low, mid, and high based on the observed range in the human tibia (48.1–101.4). The mid category corresponds to the middle third of the range (65.9–83.6), the low category includes values below 65.9, and the high category includes values above 83.6. Data are presented as mean ± 95% CI.

For the bone mimetic materials, IMI derived BMSi values of 44 for Sawbones 40 PCF (95 %CI: [43.5, 44.5]), 48 for Sawbones Skull Model (95 %CI: [45.6, 50.5 ]), 54 for Sawbones 50 PCF (95 %CI: [53.6, 54.4]), 56 for PHACON sandwich strips (95 %CI: [55.2, 56.8]), 59 for PHACON Cortical 1.2g/cc shiny surface (95 %CI: [57.0, 61.0]), 60 for PHACON Solid blocks 66Y Male (95 %CI: [58.6, 61.4]), 61 for PHACON L1-L5 66Y Male (95 %CI: [59.5, 62.5]), 64.5 for PHACON L3-L4 spine 66Y Male (95 %CI: [63.8, 65.2]), 65 for PHACON Cortical plug 1.94g/cc (95 %CI: [62.2, 67.8]), 69 for PHACON Cortical 1.2g/cc pink surface (95 %CI: [67.8, 70.2]), 74 for Stratasys Basic (95 %CI: [73.1, 74.9]), 92 for BoneSim Cortical bone 1800 (95 %CI: [89.5, 94.5]) and 93 for Sawbones 102 PCF 1.64g/cc (95 %CI: [91.6, 94.4]) (Fig. 1).

Mimetic materials within the low category included Sawbones 40 PCF, Sawbones Skull Model, Sawbones 50 PCF, PHACON sandwich strips, PHACON Cortical 1.2g/cc shiny surface, PHACON Solid blocks 66Y M, PHACON L1-L5 66Y M, PHACON L3-L4 spine 66Y M, and PHACON Cortical plug 1.94g/cc. Mimetic materials in the mid category included PHACON Cortical layer 1.2g/cc pink surface and Stratasys Basic. Mimetic material in the high category included BoneSim Cortical bone 1800 and Sawbones 102 PCF 1.64g/cc (Table 2).

**Table 2:** Categorization of bone mimetic materials into material strength categories.

Bone Mimetic Material Strength Reference		
Low	Mid	High
♦ Sawbones (40 PCF) ♦ Sawbones (Skull Model) ♦ Sawbones (50 PCF) ♦ PHACON (sandwich strips) ♦ PHACON (Cortical 1.2g/cc shiny surface) ♦ PHACON (Solid blocks 66Y M) ♦ PHACON (L1-L5 66Y M) ♦ PHACON (L3-L4 spine 66Y M) ♦ PHACON (Cortical plug 1.94g/cc)	♦ PHACON (Cortical 1.2g/cc pink surface) ♦ Stratasys (Basic)	♦ BoneSim (Cortical bone 1800) ♦ Sawbones (102 PCF 1.64g/cc)

## 4 Discussion

This study provides the first objective stratification of commonly used bone mimetic materials, animal, and human cadaveric bone based on measures of material strength. BMSi, measured via the OsteoProbe device, provided a unified scale that accommodated the spectrum of materials tested. Most importantly, these values, measured using the same modality, can be directly compared with values obtained from living human bone. These comparisons are critical for selecting the appropriate material for a specific research context. Accordingly, the synthetic materials were stratified into categories of Low, Mid, and High tertiles based on their BMSi, providing the ability to compare these materials to each other. Furthermore, the application of OsteoProbe to human cadaveric bone demonstrated its adaptability to stratify bone material quality across a spectrum of anatomical structures.

The human cadaveric values exhibited a wide range of BMSi across sex and anatomical site. For example, the female spine average BMSi was 64.2 which would most align with the softest bone mimetic materials such as the Sawbones 50 PCF or PHACON Solid Blocks 66Y M. In contrast, the male cranium BMSi was 95 and would be most accurately modeled by the hardest bone mimetic materials such as the Sawbones 102 PCF or BoneSim cortical bone analog 1800. Consistent with clinical experience male BMSi values in cadaver radius, femur, and spine, were greater than female for the same anatomical site indicating the male cadaver sites had higher material strength.^35^ Additionally, the femur had higher BMSi values than the radius followed by the spine. Perhaps most notably, several bone mimetic materials exhibited BMSi values that are inconsistent with their intended and marketed use. For example, the Sawbones Skull Model, advertised as a mimetic for hard cranial bone, ranked the lowest in BMSi compared to other materials (95 %CI: [45.5, 50.5]). This comparison may be helpful to selecting the right material that for the intended mechanical application. This study further elucidates the extent to which animal bones model the mechanical behavior of human cadaveric bone. For example, the average porcine spine BMSi was 87 while the average male spine BMSi was 68.6 and 64.2 for females. Similarly, the ovine skull BMSi was 75 while the male cadaver cranium BMSi was 95. Given these differences in material strength measured through IMI between matched anatomical sites, animal models should be used cautiously to represent the mechanical properties of human bone.

Previous work has also quantified the material strength of industrial plastics that span the BMSi range observed in living human bone.^32^ The categorization proposed in this study can be applied to these plastics for expanded material selection. Plastics falling into the low BMSi category include UHMW, HDPE, Polystyrene, and ABS; those in the mid category include Noryl PPO, Cast Nylon, Polyester, Extruded Nylon, Rexolite, and Delrin; and those in the high category include Ultem PEI, PEEK, Torlon PAI, and PMMA. This alignment reinforces the applicability of the current classification framework to a broader class of synthetic materials with well-characterized mechanical behavior.

This reference of bone mimetic material, animal, and human cadaver material strength has great potential to improve orthopedic training and research. As bone mimetic materials are widely used to ethically and conveniently model the mechanical behavior of bone, it is vital that the selected bone mimetic material accurately represents the clinical scenario. In the surgical education context, tactile feedback and correct assessment of bone strength are necessary to avoid complications. Using accurately matched bone mimetic materials for surgical training could improve resident education. Additionally, medical devices are regularly tested on bone mimetic materials during development and safety validation. Testing these devices on realistic bone mimetic materials could improve translation to clinical use. As IMI testing through the OsteoProbe continues to expand, additional bone mimetic materials and human anatomical sites can be included into this material strength categorization convention to further improve material selection.

## 5 Conclusion

OsteoProbe provides an objective, efficient, and reproducible tool for stratifying the mechanical performance of materials commonly used in orthopedic research and surgical education. This capability fills a longstanding gap by replacing subjective or manufacturer-provided assumptions with empirical data. Given that the fracture toughness and strength of bone vary widely in the clinical population, it is essential for clinicians and orthopedic researchers to select appropiate bone mimetic materials.

By providing direct comparability to human bone reference data, OsteoProbe facilitates more realistic material selection for biomechanical testing and ensures that cadaveric specimens are appropriately matched to educational and research goals. This work supports the broader adoption of impact microindentation as a standardized screening tool for materials used in orthopedics.

## Guardian/patient's consent

None.

## CRediT authorship contribution statement

Lucas R. Budd: Conceptualization, Formal Analysis, Data Curation, Writing - Original Draft, Writing - Review & Editing, Visualization. Rachana S. Vaidya: Conceptualization, Formal Analysis, Data Curation, Writing - Review & Editing. Babak Jahani: Conceptualization, Formal Analysis, Investigation. Saideep Nakka: Conceptualization, Methodology, Investigation, Data Curation, Resources, Writing - Review & Editing. Alexander Proctor: Conceptualization, Methodology, Writing - Review & Editing. Peter T. Burks: Conceptualization, Methodology, Writing - Review & Editing. Blake K. Montgomery: Formal Analysis, Writing - Review & Editing. Simon Y. Tang: Conceptualization, Writing - Review & Editing, Formal Analysis, Supervision

## Ethical statement

None.

## Funding/sponsorship

Research reported in this publication was supported by the National Institutes of Health R44AG071034 (Awarded to Peter Burks, Ph.D./Active Life Scientific, and to Simon Tang, Ph.D./Washington University in St Louis). The content is solely the responsibility of the authors and does not necessarily represent the official views of the National Institutes of Health. The authors have no other competing interests to declare that are relevant to the content of this article.

## Declaration of competing interest

Employees of Active Life Scientific, Inc. contributed to the study's design, data collection, analysis, interpretation, and article preparation. In addition, the company supported the submission of the article.
